# The Use of Auto-Titrating Continuous Positive Airway Pressure (AutoCPAP) for Obstructive Sleep Apnea Syndrome in Children with Obesity

**DOI:** 10.3390/children8121204

**Published:** 2021-12-19

**Authors:** Sarah Benke, Caroline U. A. Okorie, MaryAnne A. Tablizo

**Affiliations:** 1 Department of Pediatrics, Valley Children’s Hospital, Madera, CA 93636, USA; 2 Division of Pediatric Pulmonary and Sleep Medicine, Stanford Children’s Health, Lucille Packard Children’s Hospital, Palo Alto, CA 94304, USA; cokorie@stanford.edu (C.U.A.O.); MTablizo@valleychildrens.org (M.A.T.); 3 Division of Pediatric Pulmonary and Sleep Medicine, Valley Children’s Hospital, Madera, CA 93636, USA

**Keywords:** pediatric sleep apnea, sleep disordered breathing, obstructive sleep apnea, positive airway pressure, auto-titrating continuous positive airway pressure, obesity

## Abstract

Background: Positive airway pressure can be an effective and safe therapy for children with obstructive sleep apnea syndrome (OSAS). Few studies have assessed the safety and efficacy of autoCPAP in pediatric patients with obesity. Methods: This was a retrospective chart review of children with obesity (Body Mass Index (BMI) > 99th percentile), ages 2–18, diagnosed with OSAS (Obstructive Apnea-Hypopnea Index (OAHI) > 1/h) and used autoCPAP with 30-day adherence. Exclusion criteria included patients with complex comorbidities. Adherence was defined as autoCPAP use ≥4 h/night for at least 21/30 days. Baseline PSG OAHI was compared to the AHI from the 30-day autoCPAP compliance report. We also compared autoCPAP 30-day 95th percentile pressures with the pressures from PAP titration. Results: The study included 19 children, ranging 5–15 years old. The median BMI was 99.6th percentile and average adherence was 25/30 nights with mean of 7.3 h/night. The median OAHI was 12.3/h on baseline PSG and the 30-day autoCPAP download AHI decreased to 1.7/h. No adverse outcomes were identified. The average difference between 95th percentile autoCPAP pressure and PAP titration pressure was 0.89 cmH20. Conclusion: Our study suggests autoCPAP is effective and safe for the treatment of OSAS in pediatric patients with obesity. Using autoCPAP may reduce delays in treatment. Additional research is needed to verify the long-term effectiveness of autoCPAP in this population.

## 1. Introduction

Obstructive sleep apnea syndrome (OSAS) affects an estimated 2–4% of the general pediatric population [1]. The causes of obstructive sleep apnea in children can include adenoid and/or tonsillar hypertrophy, narrow orofacial bone shape and tongue based collapse [2]. Obesity is also a well-established risk factor for OSAS among both children and adults, and it is estimated that up to 60% of children with obesity have OSAS. Unfortunately, the rates of obesity worldwide continue to increase, with a younger and younger age at presentation [3,4,5,6]. Therefore, there is concern that the rates of OSAS among children will also increase accordingly.

It is important to ensure that those patients who are already affected with obesity and OSAS are adequately screened, diagnosed and treated in order to maintain good health and avoid any of the associated disease. Untreated OSAS in children can lead to neurocognitive issues, behavior problems, difficulties in school, poor growth and increased cardiovascular risks [7,8,9,10]. OSAS has also been associated with systemic inflammatory marker elevation, and metabolic abnormalities such as insulin resistance, dyslipidemia and disrupted visceral fat deposition [11,12]. One study demonstrated that children with OSAS may have more than a six-fold increase in risk for metabolic syndrome compared to their counterparts without OSAS [5,11,12]. Obesity is thought to contribute to the pathophysiology of OSAS in multiple mechanisms, including increased fat deposition in the upper airway leading to a reduced lumen size as well as increased airway collapsibility [11,13]. It can also lead to possible restrictive lung physiology, thereby increasing the patient’s risk for hypoxemia and/or hypoventilation [5,11,14]. Additionally, studies have shown that sleep disruption and OSAS are themselves risk factors for obesity [11,15,16]. This demonstrates the unfortunate compounding relationship between OSAS and obesity and the way one acts as a risk factor for the other. Obesity seen in childhood typically persists into adulthood leading to increased risk for various chronic diseases associated with obesity, including cardiovascular disease and diabetes [9].

The recommended first-line treatment for pediatric patients is tonsillectomy and adenoidectomy (T&A) in patients with adenoid and tonsillar hypertrophy [17]; however, the incidence of residual OSAS after surgical intervention is not insignificant. In some studies, this number has been estimated up to 49% in pediatric patients with OSAS and obesity [17], whereas other studies in the literature have demonstrated a wider range of 33–76% [4]. Because of this, other interventions should be considered in this population. Continuous Positive Airway Pressure (CPAP) therapy is a treatment for those with OSAS, who are not surgical candidates, or who have residual OSA despite T&A [17,18]. CPAP has been shown to be effective and safe in children [19]. The recommended use of CPAP in children is fixed-pressure CPAP, determined during the PAP titration obtained during overnight polysomnography (PSG). Conventional CPAP uses a set pressure (by centimeter of water pressure) to provide a pneumatic stent to maintain an open airway. However, the challenges of poor adherence and long waits for PAP titration studies have motivated physicians to consider newer technologies such as auto-titrating CPAP (autoCPAP) for the pediatric population [19,20,21]. An autoCPAP uses an algorithm to adjust pressures to meet the specific pressure demand to overcome obstructive breathing events detected by the machine.

AutoCPAP is widely used in the adult population in place of in-laboratory PSG titration studies and has been shown to effectively decrease (Apnea-Hypopnea Index) AHI and improve symptoms related to OSA. It can provide variable pressure delivery to accommodate detected changes in flow limitation in the upper airway [21,22]. The adjustment to variable pressures in response to the patient’s needs may improve comfort and play a role in better adherence to therapy [22]. One study showed that it was effective and overall well tolerated in children within an attended setting and that it could be considered as an alternative treatment for sleep-related breathing disorders in pediatric patients [22]. However, studies of pediatric-patient use in the unattended home setting are limited, with a few studies showing efficacy and safety in pediatric patients [23,24]. The medical literature lacks studies exploring the use of autoCPAP in children, specifically those with obesity. The aim of our study was to evaluate the safety and efficacy of autoCPAP as treatment for OSAS among children with obesity.

## 2. Materials and Methods

### 2.1. Study Design

A retrospective chart review was performed for children with obesity using autoCPAP at Valley Children’s Hospital between May 2016 to April 2021 (four patients before 2018 and the remaining from 2019 to present). The study was approved by the Valley Children’s Hospital institutional review board.

### 2.2. Inclusion and Exclusion Criteria

Inclusion criteria were as follows: patients with OSAS and obesity, ages 2 to 18 years without significant comorbidities. OSAS was defined as an obstructive apnea-hypopnea index (OAHI) with more than one event per hour, obtained by in-lab diagnostic polysomnography using SomnoStar sleep system (PSG). PSG was scored, based on the most recent American Academy of Sleep Medicine (AASM) scoring rules. Obesity was defined as body mass index (BMI) at or above the 95th percentile. Patients were also required to have autoCPAP use greater than or equal to four hours per night for greater than or equal to 21 days out of a total 30-day period. The adherence criteria was based on adult health-insurance guidelines for compliance, which is defined as use of CPAP greater than or equal to four hours per night for greater than 70% of a given time frame (21 days out of a 30-day period). Pediatric adherence criteria are not established. Exclusion criteria were patients with complex comorbidities, including unrepaired congenital heart disease, chromosomal anomalies, and neuromuscular disorders.

### 2.3. Data Collection

We then accessed the Cloud-based PAP compliance and therapy monitoring software from ResMed AirView to determine which patients were compliant to therapy for at least a 30-day period, using the criteria for adherence noted above. Among patients who had been adherent to therapy, the baseline PSG, PAP titration study and medical records were reviewed. All patients that met criteria were included in the study.

### 2.4. Data Analysis

We compared baseline polysomnography OAHI with the autoCPAP derived 30-day mean OAHI, reported via the cloud-based therapy data system. In order to compare the autoCPAP 95th percentile pressure to the in-lab PSG titration recommended pressure, we determined the mean difference between the autoCPAP-derived 95th percentile pressure and the PSG PAP titration pressure.

## 3. Results

**Figure 1:**
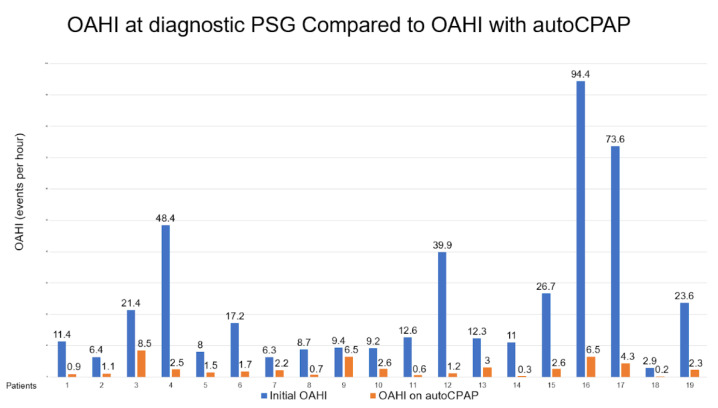
Demonstrates a significant decrease in obstructive AHI in patients after initiation of autoCPAP.

There were 19 children who met the criteria aged from 5 to 15 years old with adherent autoCPAP use. The median BMI was in the 99.6th percentile. There were 15 patients that met criteria for severe obesity with BMI greater than or equal to the 99th percentile. Average autoCPAP use was 7.3 h per night, for an average of 25 days in a 30-day period. Each patient’s OAHI was obtained from their initial baseline polysomnography and compared to their autoCPAP 30-day compliance and therapy download. The comparison for these values can be seen in Figure 1. The median PSG obstructive AHI was 12.3 events per hour, whereas the median obstructive AHI while on autoCPAP was 1.7 events per hour, indicating a substantial decrease in events with autoCPAP use.

The cloud-based PAP compliance and therapy monitoring software-downloaded reports included the 95th percentile pressure from the patients’ autoCPAP use for 30 days. This value indicates that 95% of the time spent on autoCPAP is at this pressure or below. These pressures were compared to the PAP pressure settings recommended from the in-lab PSG PAP titration (Table 1) for those patients with available PAP titration study. The median age for these patients with PAP titration studies was 10.5 (5–15) years with a median BMI of 99.55% with 9/12 of the patients with BMI > 99th percentile. The 95th percentile pressure values were obtained from the most recent compliant 30-day period autoCPAP download and compared to the pressure obtained from the PAP titration study. The time period between the titration study and the downloaded compliant period ranged from 1–7 months for most patients, but for three of the patients the time period was greater than a year. There were seven patients in total who were not able to return to the sleep lab for PAP titration or their PAP titration study was pending at the time of the evaluation. When comparing the pressure obtained during in-lab PSG CPAP titration to the Cloud-based autoCPAP 95th percentile pressure, the majority of patients (9/12) had a difference of less than 2 cmH20, whereas three patients had a difference of 2 or 2.1 cmH20. The mean cmH20 difference was 0.89 (range 0.1–2.1). There were no adverse outcomes identified upon chart review.

## 4. Discussion

The results from this study demonstrate that the use of autoCPAP among children with OSAS and obesity can reduce the number of obstructive apnea–hypopnea events. OSAS was defined as an OAHI of more than 1 event per hour, per the International Classification of Sleep Disorders by the American Academy of Sleep Medicine [25]. Our study showed that the median OAHI was reduced from the baseline PSG in patients who are adherent with autoCPAP use. In our study, autoCPAP was not only effective in the treatment of mild to moderate OSAS, but also showed a significant reduction of OAHI in severe OSAS. These findings are consistent with other studies showing an improvement of OSAS after in-laboratory PSG PAP titration, with a significant decrease in OAHI and even the complete resolution of OSAS in pediatric patients [24]. Khaytin et al. demonstrated that children in their study who used autoCPAP showed an improvement of OSAS with a decrease in OAHI from a median of 18.4 events per hour to 1.0 events per hour [21]. Another study by Palombini et al. found similar results in their study, demonstrating that autoCPAP use can resolve OSAS in pediatric patients [22].

Given the growing prevalence of obesity among the pediatric population, this is a population that will need more and more attention. Effectively treating OSAS in pediatric patients with obesity should be considered as part of the overall medical treatment plan for these patients. In adult patients with OSAS and obesity, there is a correlation in the reduction of visceral fat (as measured by abdominal computer tomography imaging) after CPAP treatment [11]. While these are adult studies, there is evidence to suggest that effective PAP treatment of OSAS can have direct adjunct effects with weight loss efforts and interventions.

Treatment with autoCPAP for OSAS can provide a wide range of pressures to facilitate adjustments for growth in children, weight changes, increase upper airway resistance against allergies and respiratory infections, and positional changes [22]. The accommodation of autoCPAP for growth is an important consideration for treatment, as their positive pressure requirement may change as the child grows. Outpatient monitoring is recommended with careful evaluation of cloud-based therapy and compliance data [22]. One study in the adult population showed that autoCPAP can be more comfortable for the patient compared to a standard fixed CPAP [22], due to the ability of the device to adjust pressure requirements based on the patient’s airflow. A randomized control trial comparing adherence and efficacy between autoCPAP and fixed CPAP determined that 65% of adult patients (13/20) preferred autoCPAP treatment, and that there was a trend towards lower leakage with autoCPAP [26]. Further research is required to determine its role in treatment and to identify barriers to adherent autoCPAP use in pediatric patients who are obese.

An abstract by DelRosso et al. found that autoCPAP use was well tolerated in 11 pediatric obese patients with adherent days of use ranging (use of 4+ h a night) from 53–100%, with an average of 5.5 h per night [23]. In our study, the average adherent autoCPAP use was for 83% of the days within a 30-day period, with a mean of 7.3 h of sleep per night. Our study was able to compare the required pressure during autoCPAP use and the PAP titration findings. However, additional research is still needed to determine if these effects are evident in a larger pediatric population with obesity.

Our study demonstrates that autoCPAP-derived pressures correlate with the titration PSG-derived pressures. The recommended PAP pressures obtained during in-lab PAP titration PSG were ≤2.1 (with six of the patients having ≤1 cmH20) pressure with a mean difference of 0.89 (0.1–2.1) when compared to the 95th percentile obtained on autoCPAP therapy download. Other studies in children have shown similar results, demonstrating a strong correlation between autoCPAP pressures and in-lab PSG PAP titration derived pressures [21,24].

Currently, the standard approach to starting children on CPAP therapy is to perform an in-lab PSG PAP titration study and then to prescribe a set CPAP pressure based on titration results [21]. In-lab PSG PAP titration has limitations, including a high cost and the long wait times at many pediatric sleep labs, leading to delays in treatment. There is also a risk of study failure due to the intolerance of PSG leads or inability to sleep outside of the home environment. The initiation of autoCPAP, prior to PAP titration, can help to avoid delays in treatment and allow for desensitization to occur in the comfort of home prior to PAP titration study, which can improve tolerability to the use of a PAP machine and the accuracy of the PAP titration study. Using autoCPAP may be particularly useful during times when PAP titrations are especially difficult to obtain, such as during a global pandemic or when patients lack reasonable access to a sleep laboratory. In the setting of the COVID-19 pandemic and stay-at-home orders that prevented in-lab polysomnography studies from being completed for months, the use of autoCPAP not only decreased exposures but could also reduce delays in treatment. The use of autoCPAP could help bridge the gap between access and availability of care for the pediatric population.

There were no adverse outcomes or side effects identified upon chart review during our study. This suggests that autoCPAP treatment can be both safe and effective when treating even severe OSAS in pediatric patients with obesity. Safety of autoCPAP use in the pediatric population has been established by previous studies [21,24], in an attended setting [22] as well as in children with sickle cell anemia [20]. A study by Mihai et al. determined that autoCPAP was safe and effective in reaching close to therapeutic CPAP pressure during home initiation of treatment in children [24] but the use in pediatric patients, specifically in the obese population in an unattended home setting has not been well studied. Our study contributes to the literature on the safety of autoCPAP use in the pediatric population.

Limitations of the study include that this is a retrospective study, and that the participant group is small. Some study subjects were excluded based on significant comorbidities, which makes this study useful for obese pediatric OSA patients without other complex health issues. We also studied compliance and therapy data for a 30-day period only, and did not study long term benefits and adherence. This study is also limited as it did not include patient’s satisfaction. A future study should not only include effectiveness but should also include patient’s comfort and satisfaction. Identifying barriers for adherent autoCPAP use in obese pediatric patients would be an important clinical problem to address in future studies.

## 5. Conclusions

AutoCPAP use by pediatric patients with OSAS and obesity was associated with a significant reduction in OAHI in this study. The use of autoCPAP appears safe and tolerated in this population. There is also concordance between the optimal pressures obtained from the in-lab PSG PAP titration study to the autoCPAP 95th percentile pressure. Long wait times for PSG evaluation and subsequent PAP titration studies can delay the diagnosis and management of OSAS, which can increase patient risk for associated sequelae. AutoCPAP use can mitigate these issues. Additional research is needed to establish the long-term effectiveness, safety, and tolerability of autoCPAP in children with obesity.

## Figures and Tables

**Table 1:** Compares PAP titration recommended settings with autoCPAP 95th percentile pressures in the setting of the patient’s age and BMI.

Patient Age	Patient BMI Percentile	PAP Titration (cmH20)	Cloud-Based PAP Monitoring Report 95th Percentile Pressure (cmH20)	Difference between PAP Titration Pressure to Cloud-Based PAP 95th Percentile Pressure (cmH20)
15	99.7%	10	11.5	−1.5
15	99.8%	10	9.2	0.8
9	99.8%	7	7.8	−0.8
9	99.3%	10	9.9	0.1
13	99.7%	13	11.1	1.9
5	97.2%	10	9.6	0.4
9	99.2%	12	10.0	2.0
10	97.0%	11	12.5	−1.5
11	99.4%	20	18.0	2.0
14	99.7%	10	9.9	0.1
12	95.4%	9	10.0	−1.0
9	99.9%	14	11.9	2.1

## Data Availability

Restrictions apply to the availability of these data. The data are not publicly available due to patient privacy.
